# Textile Pressure Sensor Made of Flexible Plastic Optical Fibers

**DOI:** 10.3390/s8074318

**Published:** 2008-07-25

**Authors:** Markus Rothmaier, Minh Phi Luong, Frank Clemens

**Affiliations:** 1 Empa, Swiss Federal Laboratories for Materials Testing and Research, Laboratory for Protection and Physiology, Lerchenfeldstrasse 5, 9014 St. Gallen, Switzerland; 2 Empa, Swiss Federal Laboratories for Materials Testing and Research, Laboratory for High Performance Ceramics, Ueberlandstrasse 129, 8600 Duebendorf, Switzerland

**Keywords:** thermoplastic silicone, weaving, sensor array, light coupling between fibers

## Abstract

In this paper we report the successful development of pressure sensitive textile prototypes based on flexible optical fibers technology. Our approach is based on thermoplastic silicone fibers, which can be integrated into woven textiles. As soon as pressure at a certain area of the textile is applied to these fibers they change their cross section reversibly, due to their elastomeric character, and a simultaneous change in transmitted light intensity can be detected. We have successfully manufactured two different woven samples with fibers of 0.51 and 0.98 mm diameter in warp and weft direction, forming a pressure sensitive matrix. Determining their physical behavior when a force is applied shows that pressure measurements are feasible. Their usable working range is between 0 and 30 N. Small drifts in the range of 0.2 to 4.6%, over 25 load cycles, could be measured. Finally, a sensor array of 2 × 2 optical fibers was tested for sensitivity, spatial resolution and light coupling between fibers at intersections.

## 1. Introduction

New applications in the field of so-called smart textiles for health monitoring are having a high demand of new techniques to successfully miniaturize and embed electronics, optics and sensors into fabrics and garments [1-4]. The benefit of close to the body measurements are numerous e.g. enhanced comfort and ease of movement for the wearer, reduction of loose connecting wires between sensors, electronic circuits and energy sources. Textile integrated sensors could measure a large variety of variables, e.g. physical dimensions like pressure, stress and strain [5-10] applied to the textile or biomedical dimensions such as heart rate, electrocardiogram (ECG), sweat rate and sweat composition (salts, pH), respiration rate or arterial oxygenation (SpO_2_) of the monitored subject [11-16]. Most of these sensors are based on microelectronic devices or conductive polymers which are integrated into the fabric structure, or are part of the fibrous structure themselves. Optical fibers made of polymeric materials have the advantage of high flexibility an low stiffness compared to glass fibers, therefore they are receiving more and more attention in the field of smart textiles and will complement electrical wires and sensors in the near future. A couple of advantages make their application very attractive: they produce no heat, they are insensitive to electromagnetic radiation and they are not susceptible to electrical discharges. Several types of textile sensors already have been developed using optical fibers based on grating or microbend principles [13, 15, 17-21].

**Figure 1.**
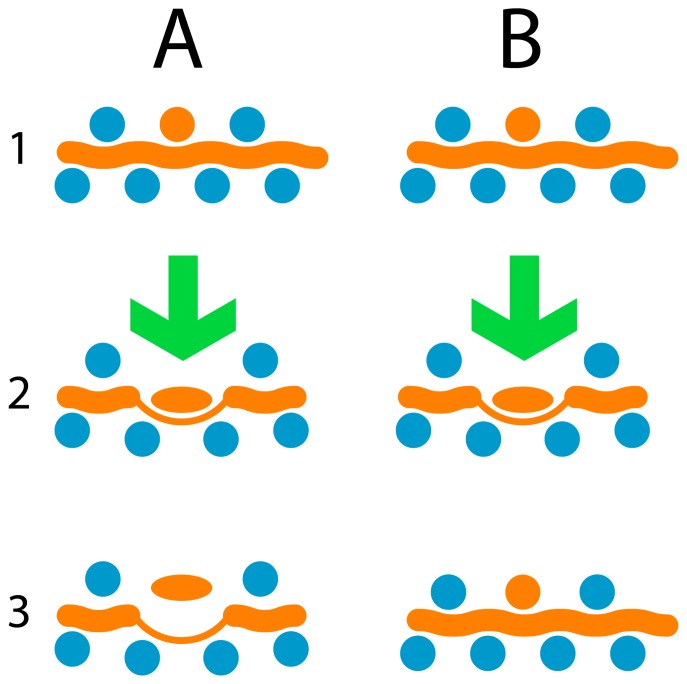
Functional schematic of a pressure sensor (optical fibers in orange, textile fibers in blue) where POF are not reversibly bent and squeezed (case A) or for a fully elastic case (B).

However, standard plastic optical fiber (POF) materials like polymethylmethacrylate, polycarbonate and polystyrene are rather stiff compared to standard textile fibers and therefore their integration into textiles usually leads to stiffen of the woven fabric and the textile touch is getting lost. Alternative fibers with appropriate flexibility and transparency are not commercially available; few examples are mentioned in the literature, among them silicones [22, 23]. The manufacture of these flexible silicone POF has been laborious, because two-component thermoset materials have been used. These materials had to be mixed first, and later filled into adequate soft tubing materials and cross-linked inside finally (by heat or catalysts). This method allows only the production of rather short fiber lengths with large diameters. Furthermore, the procedure is error-prone to air bubbles, material shrinkages and tubing inhomogeneities. To overcome these disadvantages, we are reporting in this paper about our approach to manufacture silicone fibers made of thermoplastic material and their application as textile integrated pressure or touch sensors. Geniomer, a rather new block copolymer made up of soft and hard segments (siloxane/urea) was chosen, due to its elastomeric properties, suitable optical transparency and flexibility – combined with the thermoplastic characteristic induced from urea units. Our goal was to establish a new fiber optic sensor type, where the fiber itself shows a fully reversible elastic deformation (see Fig. 1). The application of pressure would lead to a squeezed fiber profile where light could not propagate further, or at least part of it would leave the light guide (back reflection, side emission). By integrating several fibers into a fabric sensor arrays could be established as basic touch transducers (on/off switch, or for input devices like keyboards) or more sophisticated pressure and activity monitors in e.g. beds for geriatric care (nursing homes), decubitus prevention of patients (beds or wheel chairs), prevention of sudden infant death syndrome, but also for sports (rehabilitation) and many industrial applications.

## 2. Experimental Section

### 2.1. Extrusion and coating of silicone fiber

The core material of the flexible silicone fiber consists of Geniomer 200 (Wacker AG, Germany), a polysiloxane-urea-copolymer with a polysiloxane content of > 90%. The polymer was used as supplied with no further treatment. The melt flow index (MFI) has measured 17.78 ± 0.52 g/10min (n = 3) at 1 kg and 170°C (Wacker datasheet: 170°C, 2.16 kg, 110-150 g/10min), and a melting temperature (T_m_) of 171.31 ± 1.52°C has been determined by differential scanning calorimetry (n = 3; Wacker datasheet 160-190°C).

**Figure 2.**
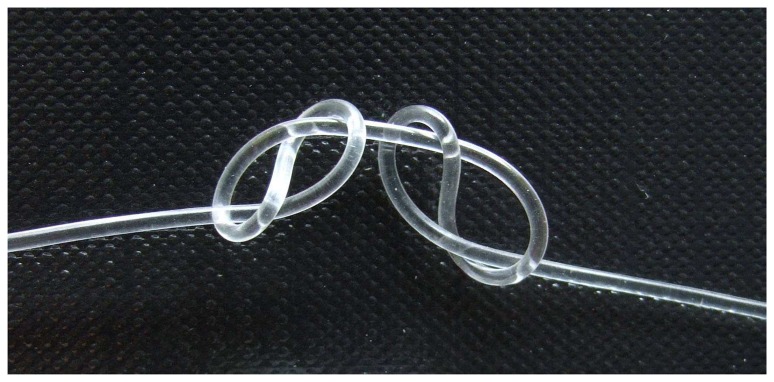
Extruded silicone fiber of 0.51 mm core diameter.

The material has a shore A of 55-65 with elongation at break > 400% (Wacker datasheet). The supplied pellets have been dried in an oven at 80°C for 55 hours before extrusion (water content < 0.3%; Wacker datasheet). The fibers were extruded using a capillary rheometer RH7 Flowmaster (Bohlin Instruments GmbH, Germany): process temperature 167°C, die diameter 0.5 mm, orifice length 8 mm, entry angle 180°. Ramp speed between 0.5 and 3.0 mm/min (consequential force between 1.87 and 3.18 kN) delivered four individual fibers of 0.21 ± 0.06, 0.38 ± 0.05, 0.51 ± 0.03 and 0.98 ± 0.03 mm diameter (n = 8). Their surface was relatively sticky, so they were stored on paper drums (loops not touching each other). To prevent them from dust particles, they were stored additionally inside antistatic bags. A low refractive index coating material (THV 220, *n* = 1.36; Dyneon, Germany) was later applied by dip coating the fibers in 5 wt% acetone solution (23°C). The coating thickness was calculated to 16 ± 3 μm by gravimetric analysis (n = 3). After the dip coating with THV polymer they lost their sticky behavior. The thinnest fibers (core diameter 0.21 and 0.38 mm) were not considered for further investigations due to their poor thickness homogeneity. Fig. 2 shows a sample of the 0.51 mm fiber having two interloops, that can reversibly been contracted and opened without visible damage.

### 2.2. Light attenuation measurement

Light attenuation was measured according to the cut back method [24]. The silicone fibers were glued into F-SMA connectors (Precimation, Switzerland) with standard epoxy; fiber ends were cut with a scalpel (no polishing is possible, due to the rubbery character). A medicinal laser LC PDT 652-2 (652 nm; AOL Medical Instruments, USA) was used as a light source, having a FD-1 fiber (Medlight, Switzerland) and a proprietary F-SMA coupler attached as an interface to the silicone fiber. The light energy was measured with an Ulbricht integrating sphere (RW-3703-2; Gigahertz Optik, Germany). Attenuation at a wavelength of 652 nm has measured 9.3 ± 0.8 and 5.5 ± 0.9 dB/m for the 0.51 and 0.98 mm fiber, respectively. However, we assume that at least 1 dB results from the poor quality of the untreated fiber ends [24].

### 2.2. Manufacturing of weave

**Figure 3.**
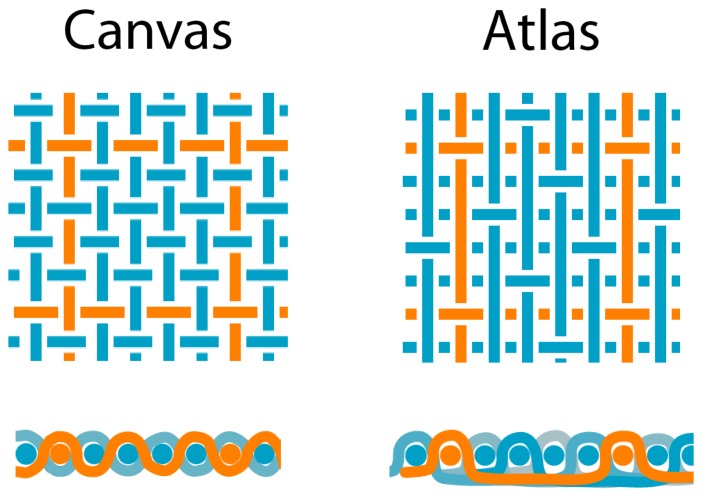
Canvas and atlas weave patterns (top and front view); blue = cotton fibers, orange = optical fibers. The atlas pattern has the weft POF always over the warp POF (top and bottom positions).

Two woven samples were produced with a hand loom (ARM AG, Switzerland) of cotton multifilament fiber of 0.75 mm (Leibundgut, Switzerland) and with the 0.51 and 0.98 mm silicone POF respectively. We have chosen a so-called atlas pattern with a 1/4 repeat (see Fig. 3) because, compared with the standard canvas pattern, only few fiber dislocations from bottom to top (or vice versa) occur – therefore a minimum light attenuation, caused by microbends within these dislocations, is expected when the fabric is in its unloaded state.

**Figure 4.**
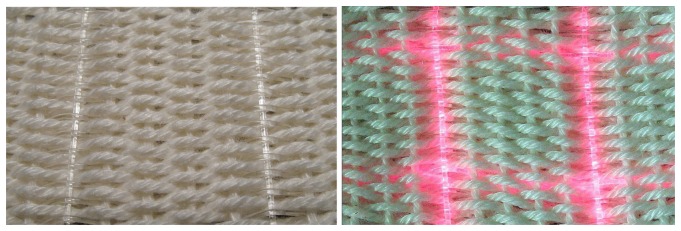
Atlas pattern weave of 0.51 mm silicone POF and cotton multifilament fibers in warp and weft direction (left picture). Four POF in a distance of 1.5 cm have been chosen to define a 2 × 2 matrix with four optical fiber intersections (right picture).

For our forthcoming experiments a 2 × 2 POF matrix has been appointed. Due to low fiber density in warp direction (one POF and four cotton fibers per 1.5 cm) two adjacent POF have been connected to the light sources and detectors. In weft direction a much higher fiber density is reached (8 POF and 32 cotton fibers per 1.5 cm), therefore only every 8^th^ POF was connected. The four POF of this 2 × 2 matrix therefore defined a square of 1.5 × 1.5 cm (see Fig. 4).

### 2.3. Measurement set-up light in-coupling and detection

**Figure 5.**
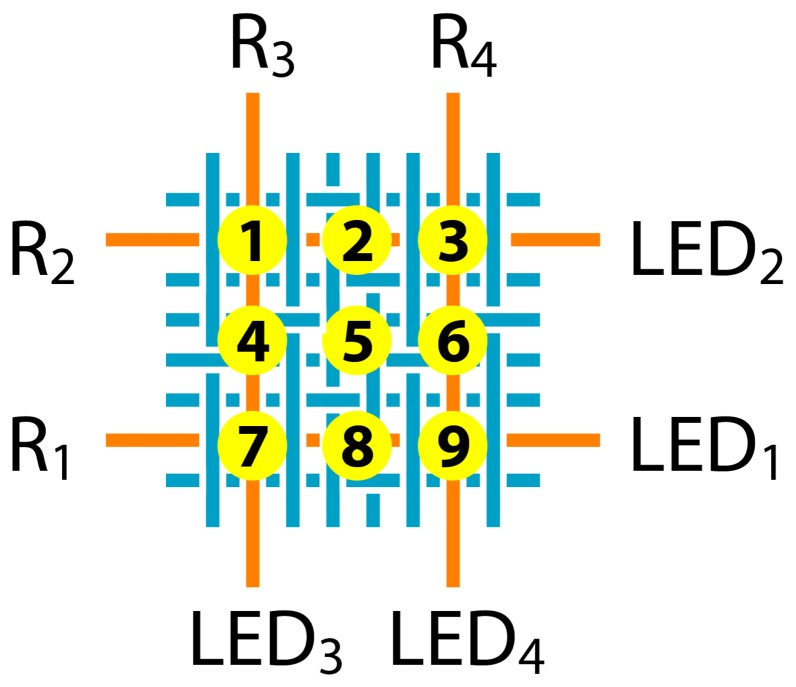
Location indices *1* to *9* (where pressure gauge head was placed). LED_x_ = light emitting diodes; R_x_ = light receiver (phototransistors). In weft direction, the graph shows only the POF (orange) which were used to define the 2 × 2 matrix (7 POF in between are not connected to a LED or R and are neglected in the graph).

A proprietary breadboard set-up has been used with four 660 nm light emitting diodes (LED; SFH 756V; Avago, USA) and four phototransistors (SFH 350V; Avago, USA) in a design according to Fig. 5. The detector signals were recorded with an analog/digital converter board (USB-6009; National Instruments, USA) at 14 bit resolution with 10 Hz data acquisition rate. Data from the converter board was transferred to a standard personal computer using dedicated software (developed with LabView 8.2; National Instruments, USA). For data analysis Origin 8 (OriginLab, USA) and SPSS 14 (SPSS, USA) has been used.

### 2.4. Force measurements

Pressure has been applied onto the fibers by means of a pressure gauge (Stoppani, Switzerland); diameter gauge head = 10 mm. The samples were placed onto a flat metal surface which was covered with a thin sheet of black cardboard. Five different experiments have been conducted (for location index see Fig. 5), with three repetitions in each case:

- measuring the relation of force versus sensor signal for 0.51 (0-20 N) and 0.98 mm fiber fabric (0-30 N) just at a single POF intersection
- measuring the signal drift behavior for both weaves, when a cyclic load (20 N) was applied
- measuring the location sensitivity of the 0.98 mm fabric when pressed at POF intersections *1*, *3*, *7* and *9* and also at non-intersections *2*, *4*, *5*, *6* and *8* (20 N)
- measuring the sensor response of the 0.98 mm weave when contacted (20 N) at locations *1*, *3*, *7* and *9* – with all four LED ON
- and measuring the same sensor response when contacted at locations *1*, *3*, *7* and *9* – with LED no. 1 and 2 ON, 3 and 4 OFF, respectively.

## 3. Results and Discussion

To investigate the sensor behavior of the woven smart fabric prototypes four different routines were setup: a) Pressure applied versus sensor signal of bottom and top fiber at an intersection (both for 0.51 and 0.98 mm weaves), b) drift behavior of sensor fabric, when pressed and released 25 times over a short time, c) signal allocation for a 2 × 2 POF matrix when two or four LED are used for irradiation, and d) signal sensitivity when pressure is applied outside POF intersections (also, for a 2 × 2 POF matrix).

### 3.1. Measurement of applied force versus sensor signal

**Figure 6.**
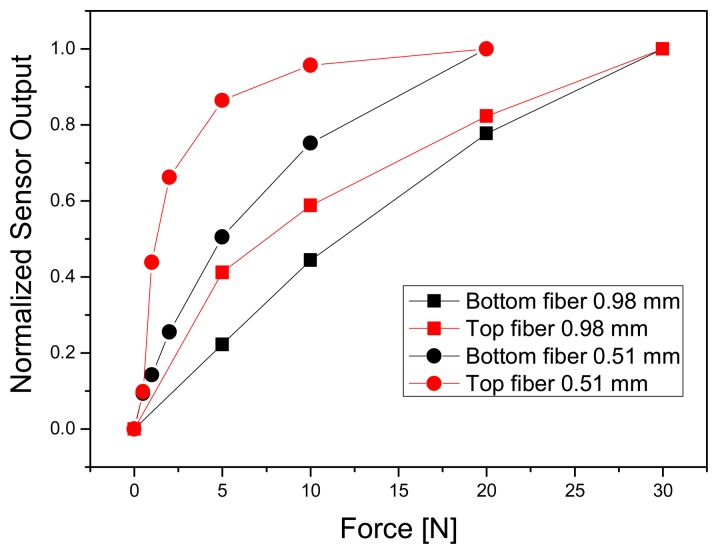
Measurement of force versus normalized sensor signal for POF fabric made of 0.51 mm diameter (round markers) and 0.98 mm (square markers) fibers, subdivided in top (red) and bottom fiber (black) response.

Measurements of applied pressure versus sensor signal were performed for the 0.51 and 0.98 mm weave, and in particular the response of POF in top and bottom position at the intersection has been studied. The comparison of 0.51 and 0.98 mm weave generally showed that thinner POF have a very sensitive response for small forces, but a small measuring range before equilibrium was reached (see Fig. 6). The 0.51 mm weave could already detect forces of 0.5 N, with a resolution (defined as three times noise level) of 0.1 N, but reached already sensor signal saturation at 10-15 N. One reason for this finding could be the limited accessibility of the thin fibers, because the surrounding textile threads will carry from a certain point on the main part of load. Thicker POF have a lower signal resolution at small forces (0.25 N), but show a practical measuring range up to 30 N (with 1 N resolution). In both cases, the top fiber showed a much pronounced sensor signal for small forces, probably due to the fact, that the top fiber bends and squeezes first when pressure is applied.

### 3.2. Measurement of drift

**Figure 7.**
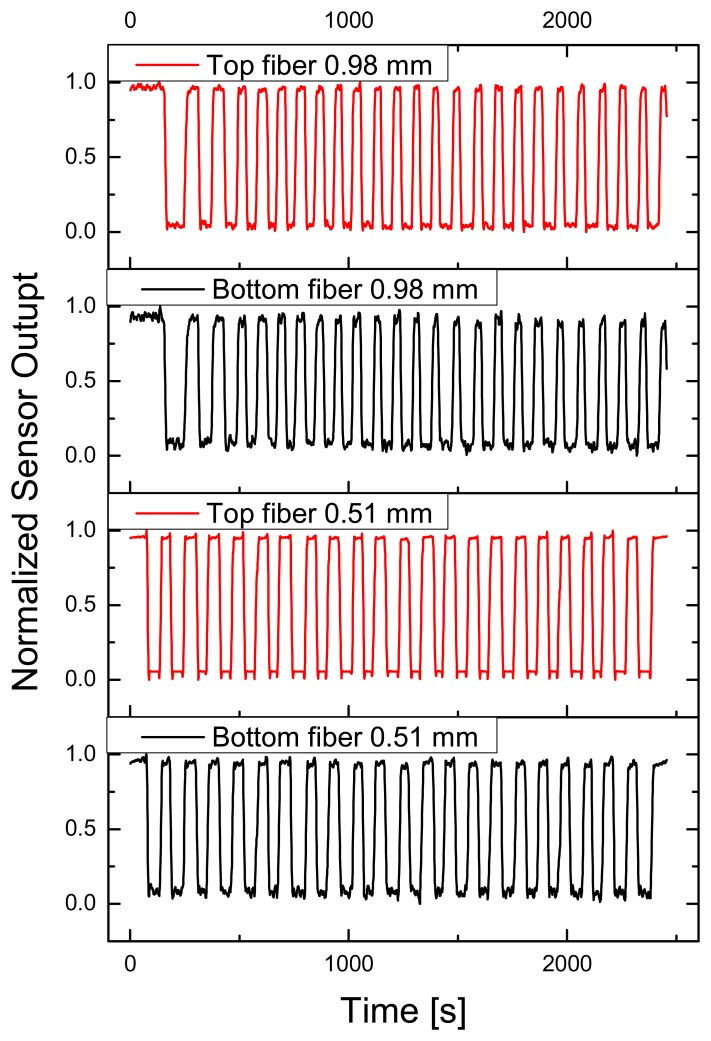
Drift behavior of POF fabric during 25 load cycles (applied force 20 N). Top (red) and bottom fiber (black) response given for 0.51 and 0.98 mm weave.

To determine short time drift or hysteresis of the sensor, which could be the result from inelastic POF or weave properties (or a combination of both), 25 load cycles have been recorded over about 45 seconds. Elastic behavior of top and bottom fibers has been monitored, for 0.51 and 0.98 mm weaves, the results are displayed in Fig. 7 and Table 1.

Clearly there can be seen a difference between both weaves, and also between the top and bottom POF. Signal drift of top fibers is small for both weaves (when pressure applied and textile relaxed, respectively), but the corresponding bottom POF showed a significant drift. The value received for the 0.98 mm weave was 2.5 to 4.5 times higher than for the 0.51 mm weave. We assume that a possible reason for this behavior is the enhanced shielding of the thinner POF as a result of the thicker surrounding textile threads.

### 3.3. Spatial resolution

**Figure 8.**
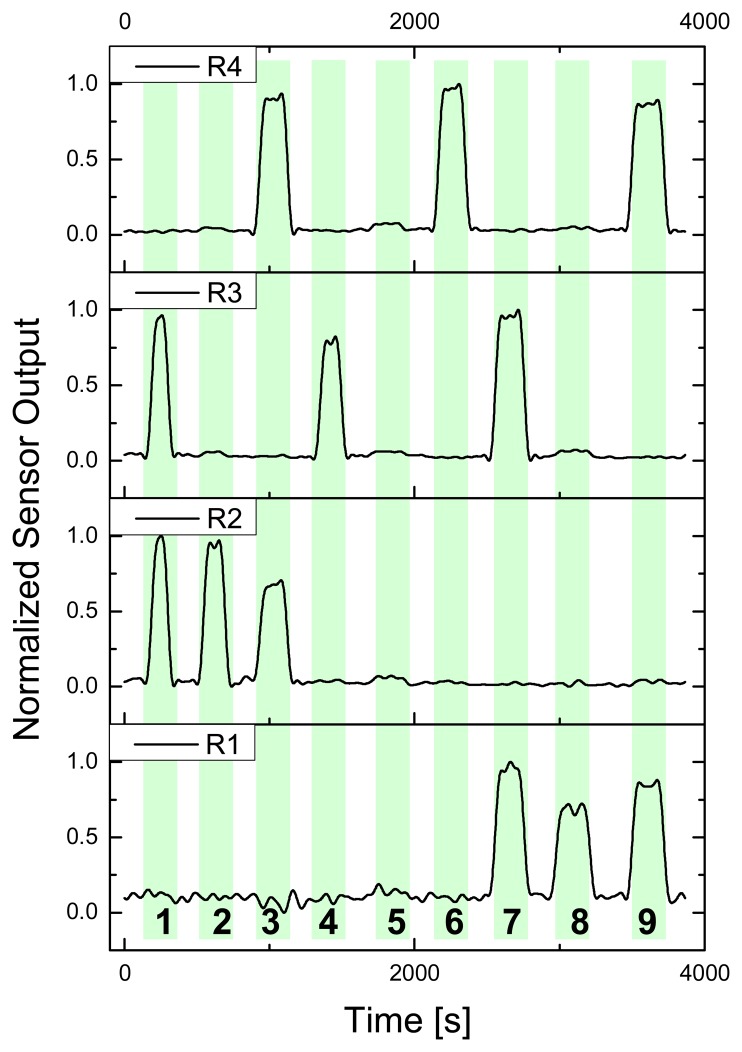
Force of 20 N applied through pressure gauge on locations *1* to *9* of the 2 × 2 POF matrix with corresponding normalized sensor signals (R_1_ to R_4_).

To quantify the spatial resolution of the POF fabric a force of 20 N was applied at 9 different fabric locations, four of them at POF intersections (*1*, *3*, *7* and *9*), four at single POF locations (POF in weft direction: *2* and *8*; POF in warp direction: *4* and *6*) and one in a POF free area (5). Fig. 8 shows an overview of the normalized sensor signals received. It is obvious that hardly any cross-sensitivity exists in the present sample (none of the interfering signals is > 5%) for all nine locations, and also no noteworthy difference for POF in weft (R_1_ and R_2_) or warp direction (R_3_ and R_4_) could be noticed. Therefore, the 2 × 2 POF matrix produced has a resolution of 9 pressure sensitive locations inside an area of 1.5 × 1.5 cm. Though, as mentioned in section 2.2, only every 8^th^ POF in weft direction was used to define the matrix – hence, an apparent potential for higher resolution is available.

### 3.4. Light coupling between fibers

**Figure 9.**
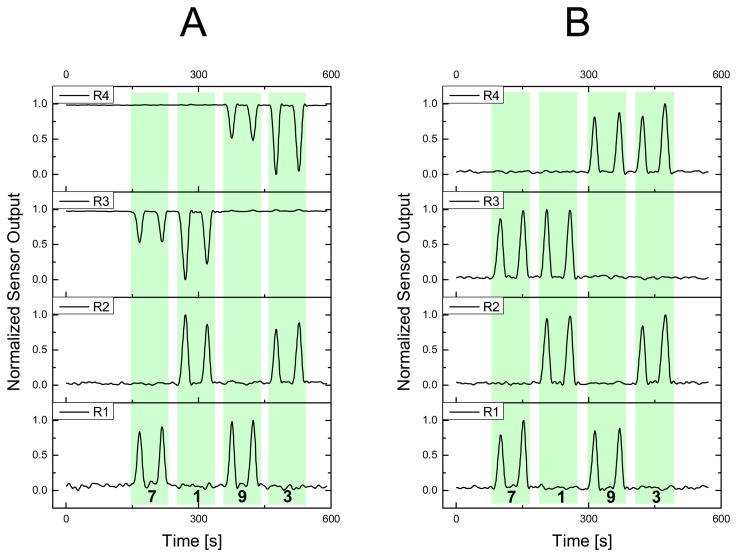
Force of 20 N applied through pressure gauge on locations *1*, *3*, *7* and *9* of the 2 × 2 POF matrix with corresponding normalized sensor signals (R_1_ to R_4_). Case A having LED_1_ and LED_2_ ON, and case B having LED_1_ to LED_4_ ON.

For an explicit allocation of where pressure has been applied not necessarily every fiber has to be connected to a light source. We found, that it is also possible to detect light decrease in weft and light increase in warp direction when pressure is applied, due to light coupling between two POF at every intersection. The following cases A and B illustrate this behavior (see also Fig. 9). In case A (LED 1 and 2 were ON; weft fibers) R_1_ and R_2_ showed a positive signal change (equals less light on the phototransistor) when POF intersections *1*, *3*, *7* or *9* were pressed. However, R_3_ and R_4_ indicated that small amounts of light have been coupled into the weft fibers (a smaller signal indicates more light on the phototransistors). Case B, where all four LED were ON, showed only decreasing light intensities at all detectors as already demonstrated in the former section. The amount of light transferred between POF (case A) could be increased probably by reworking the POF intersections to facilitate light emission and light reception. A potential modality would be the removal of cladding polymer (chemical, thermal or mechanical procedure), the supplement of index matching materials or optical components between the POF. Or on the other side an optimization of the textile pattern in a way, where one or both POF are more exposed to an external load (and not partially hidden inside the fabric). However, we assume that the small amount of light coupled between fibers could not be used in a quantitative way since textile structures are typically not rigidly coupled. Therefore the fiber touching points are dynamically moving and the quality of light coupling changes continuously.

## 4. Conclusion

In the present report, we have demonstrated a pressure sensitive sensor array which is based on flexible optical fibers integrated into woven textiles. At first, we developed an easy route to get new optical fibers from merchantable thermoplastic silicones. They showed excellent flexibility and could be manufactured with diameters in the range of 0.2 to 1 mm in an undemanding extrusion process. Their light transmission, compared to standard optical polymers, is poor, but sufficient for short distances. Materials with lower light attenuation are needed however for future applications. Secondly, these silicone fibers were integrated into woven fabrics and their use as pressure or touch sensors was evaluated. A 2 × 2 sensor matrix was able to clearly distinguish between forces applied with a lateral resolution of 10 mm. The design of the sensor matrix however does not allow shape recognition of an object or multi-touch sensitivity, because the sensor and the signal transmission element are combined in one entity. A more complex approach would be needed for such requirements [18, 21, 25-27].

## Figures and Tables

**Table 1.** Drift behavior of 0.51 and 0.98 mm POF weaves during 25 cycles of applied force (20 N) and complete release.

	0.51 mm Top	0.51 mm Bottom	0.98 mm Top	0.98 mm Bottom
Drift [%] pressure applied	0.6 ± 0.2	1.8 ± 0.3	0.2 ± 0.1	4.6 ± 0.6
Drift [%] no pressure	0.2 ± 0.1	0.6 ± 0.1	0.5 ± 0.2	2.8 ± 0.5
